# What Lies Within?

**DOI:** 10.5005/jp-journals-10005-1045

**Published:** 2009-04-26

**Authors:** Nidhi Gupta, Manohar Bhat, Rajesh Sharma

**Affiliations:** 1 PG Student, Department of Pedodontics and Preventive Dentistry, Jaipur Dental College, Jaipur, Rajasthan, India; 2 Professor and Head, Department of Pedodontics and Preventive Dentistry, Jaipur Dental College, Jaipur, Rajasthan, India; 3 Reader, Department of Pedodontics and Preventive Dentistry, Jaipur Dental College, Jaipur, Rajasthan, India

**Keywords:** Odontogenic fibroma, odontogenic tumor.

## Abstract

What follows is a case report of a 12 years old child who complained of gaps between teeth in the lower front region. OPG showed tooth 43 impacted and malformed with enlarged pulp chamber and a unilocular radiolucency surrounding the crown. Surgical enucleation was done under LA. Biopsy was sent for histopathological examination revealing the miracle diagnosis of "Central Odontogenic Fibroma" being a rare tumor of odontogenic tumor family and also a rare finding regard to age, site and clinical behavior of tumor is reported.

## INTRODUCTION

Central odontogenic fibroma is a rare and benign neoplasm of jaw. It is derived from mesenchymal component of odontogenic apparatus that is dental papillae, dental follicle and periodontal ligament. Clinically it is more frequently seen in children and young adult. Commonly found in mandible but in posterior regions. Radiographically, majority of COF are radiolucent with multilocular radiolucency and rarely unilocular. Lesion often contains small radiopaque flecks of varying density.

Histologically WHO type COF consists of mature cellular fibrous connective tissue with many islands of odontogenic epithelium. Osteoids, dysplastic dentin and cementum can be seen.

Case of central odontogenic fibroma^1^ and central odontogenic fibroma—granular cell variant type^2^ have been reported in the literature.

## CASE REPORT

A 12 years old male child presented to Jaipur Dental College, Department of Pedodontics and Preventive Dentistry. The chief complaint of patient was gaps between teeth in lower front region. Patient gave history of crown fracture of 42 which he had got endodontically treated.

The patient used *Neem datun* to maintain his oral hygiene and behaviorally the patient was cooperative.

Extraoral examination revealed that patient was of normal built with facial symmetry bilaterally symmetrical, oval facial form and a straight facial profile.

**Fig. 1.**
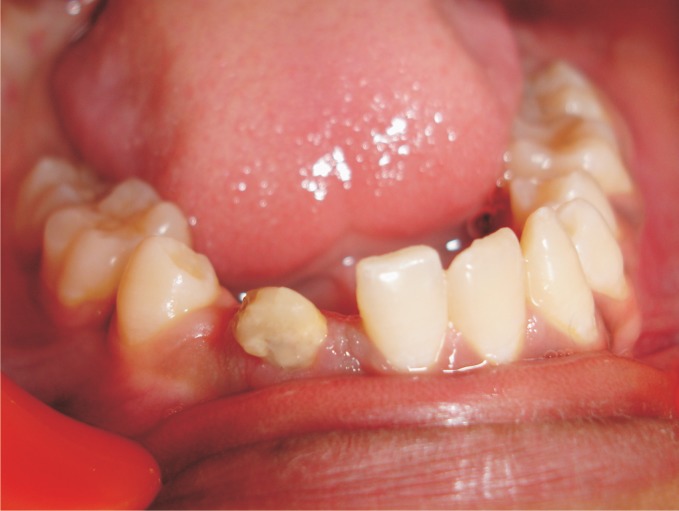
Intraoral lower arch

On Intraoral examination soft tissue appeared normal. On hard tissue examination all permanent teeth were present except 41, 43 and 45 (Fig. 1).

**Fig. 2.**
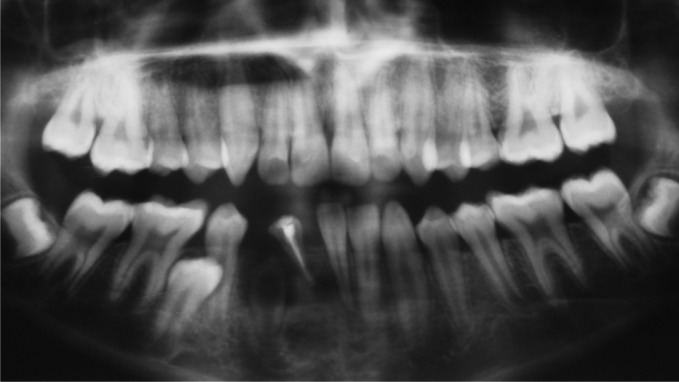
Preoperative OPG

42 showed unsatisfactory restoration and was drifting distally. Molar relation on left side was class I and on right side class III.

The patient was advised for OPG to see the missing teeth (Fig. 2).

Radiographic examination revealed:

41: congenitally missing

42: endodontically treated

45: impacted, and an unusual finding was seen in relation to 43 which was impacted and malformed with enlarged pulp chamber and radiolucency completely surrounding the crown.

At this juncture, the patient was referred to orthodontic department for further opinion regarding definitive orthodontic treatment plan and it was decided to first undergo surgical extraction of malformed 43 followed by fixed orthodontic therapy. Therefore, the treatment plan was, first the oral prophylaxis was done, then surgical extraction of malformed 43 followed by restoration of 42 followed by fixed orthodontic therapy.

## SURGICAL PROCEDURE

**Fig. 3.**
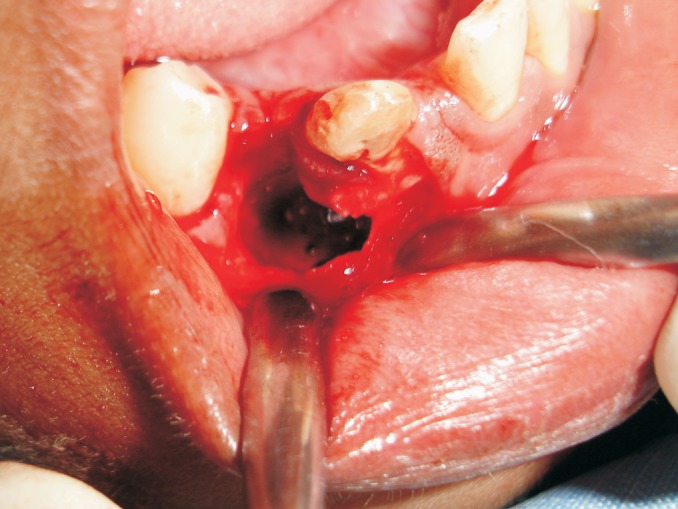
Extracted socket

The surgery was planned and consent of parents was taken. The inferior alveolar nerve block was given on right side. The incision extending from mesial of 42 to 44 was made and the envelop flap was raised and the lesion was exposed. Then, we surgically extracted 43 along with the soft tissue lesion (Fig. 3). The flap was repositioned and interrupted sutures were given.

**Fig. 4.**
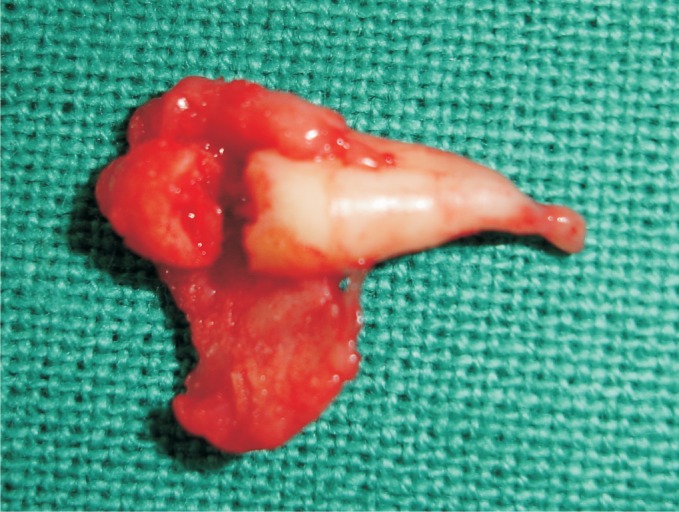
Extracted 43

Then, the specimen (Fig. 4) was sent to oral pathology department for histopathological examination.

## MICROSCOPICALLY

**Fig. 5.**
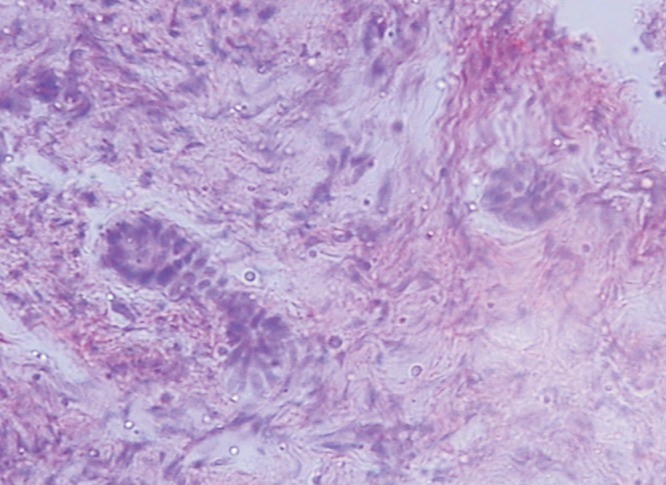
Histopathological slide 1

**Fig. 6.**
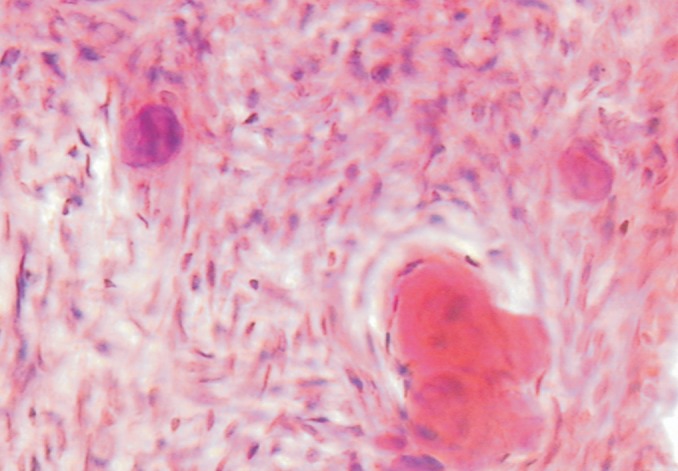
Histopathological slide 2

Soft tissue section consisted of soft connective tissue stroma with collagen fibers arranged in whorl pattern. The fibrous component varied from fibrous to myxoid. Island of odontogenic epithelium (Fig. 5) were visible all over the connective tissue stroma. Some islands appeared ameloblast like cells and stellate reticulum and some solid lacking features of odontogenic epithelium. Few islands were surrounded by eosinophilic material (Fig. 6). Numerous calcification in form of dentids and cementum visible. Fibrous capsule with strands and islands of odontogenic epithelium was seen. Dentin hypocalcified at places and showed interglobular dentin. Regular enamel space was seen. Cementum appeared normal. Pulp showed collagen fiber, blood vessels and pulp stones. Pulp stones did not resemble dentin.

Seeing the histological findings the diagnosis came as* "Central odontogenic fibroma-WHO type".*

## DISCUSSION

Central odontogenic fibroma is a rare and a benign neoplasm of jaw. It is derived from mesenchymal component of odontogenic apparatus that is dental papillae, dental follicle and periodontal ligament.^3^

Clinically, it is found in maxilla and mandible. More frequently seen in mandible in the posterior region but, in this case it was seen in anterior region of mandible.^4^

**Fig. 7.**
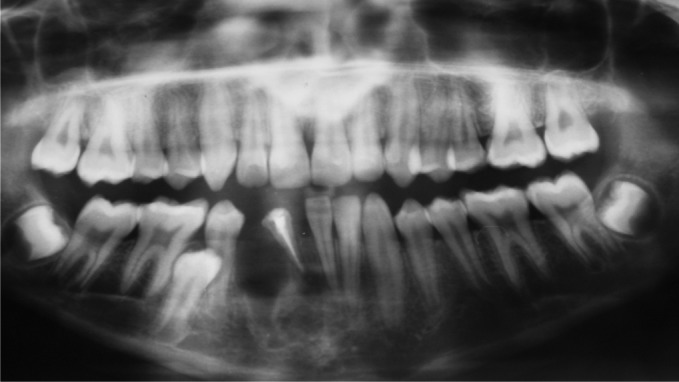
Postoperative OPG

It is asymptomatic and displacement of teeth can be seen. It is more commonly seen in children and young adults and is more prediculent in females.^5^ But in this case it was seen in a young boy.

Radiographically, majority of COF are radiolucent with multilocular radiolucency and rarely unilocular.^3^ In this case unilocular radiolucency was seen. Lesion often contain small radiopaque flecks of varying density. Postoperative OPG is shown in Figure 7.

Histologically, the WHO type consist of mature cellular fibrous connective tissue with many islands of odontogenic epithelium. Osteoids, dysplastic dentin and cementum can be seen.^6^The histopathological finding of this case were very much similar to central odontogenic fibroma (WHO type) therefore, this diagnosis was given.
